# Echocardiography, an Indispensable Tool for the Management of Diabetics, with or without Coronary Artery Disease, in Clinical Practice

**DOI:** 10.3390/medicina56120709

**Published:** 2020-12-18

**Authors:** Konstantinos Katogiannis, Dimitrios Vlastos, Foteini Kousathana, John Thymis, Aikaterini Kountouri, Emmanouil Korakas, Panagiotis Plotas, Konstantinos Papadopoulos, Ignatios Ikonomidis, Vaia Lambadiari

**Affiliations:** 1 Second Cardiology Department, ‘Attikon University Hospital’, Medical School, National and Kapodistrian University of Athens, 12462 Athens, Greece; johnythg@gmail.com (J.T.); ignoik@gmail.com (I.I.); 2 Department of Cardiac Surgery, Royal Brompton Hospital, London SW3 6NP, UK; D.Vlastos@rbht.nhs.uk; 3 Second Department of Internal Medicine, ‘Attikon University Hospital’, Medical School, National and Kapodistrian University of Athens, 12462 Athens, Greece; f.kousathana@hotmail.com (F.K.); katerinak90@hotmail.com (A.K.); mankor-th@hotmail.com (E.K.); pplotas@upatras.gr (P.P.); vlambad@otenet.gr (V.L.); 4 Echocardiography Laboratory, European Interbalkan Medical Center, 57001 Thessaloniki, Greece; papadocardio@gmail.com

**Keywords:** speckle tracking echocardiography, doppler echocardiography, stress echocardiography, coronary flow reserve, diabetic cardiomyopathy

## Abstract

Diabetes mellitus is a major factor contributing to the development of cardiovascular disease. As morbidity and mortality rates rise dramatically, when target organ damage develops pre-symptomatic assessment is critical for the management of diabetic patients. Echocardiography is a noninvasive and reproducible method that may aid in risk stratification and in evaluation of treatment effects. The aim of this review is to analyze the echocardiographic techniques which can detect early alteration in cardiac function in patients with diabetes.

## 1. Introduction

Diabetes mellitus (DM) enhances coronary atherosclerosis and impairs microcirculation leading to left ventricular (LV) dysfunction. Ramifications of DM in the coronary circulation are quite broad. DM causes development of atheromatic plaques in coronary arteries, which leads to a narrowing of intravascular lumen and to thrombus formation. On the other hand, microcirculatory impairment is caused by the toxic effect of free radicals that are formulated due to persistent hyperglycemia and might provoke arteriolar thickening, perivascular myocardial fibrosis, capillary obstruction and finally endothelial dysfunction [1].

Insulin resistance and glycemic dysregulation have detrimental effects on the systolic and diastolic function of left ventricle. Cardiac alterations in patients suffering from DM, without overt coronary artery disease, are referred as diabetic cardiomyopathy. Development of diabetic cardiomyopathy is a gradual process, which consists of the following alterations. Initially, thickness of left ventricular walls increases and left ventricular compliance declines. As a result, diastolic function is impaired and left ventricular end-diastolic pressure rises. Progressively, left ventricular volumes increase and left ventricular deformation deteriorates. Finally, heart failure syndrome occurs. As a result, diabetes has an important clinical impact in the development of cardiovascular adverse events [2].

Rubler et al. defined diabetic cardiomyopathy in 1972 [3], after studying a small population of four patients with DM, symptoms of heart failure without overt cause, in whom coronary artery disease had been excluded. According to the relevant literature, most potent mechanisms for the development of diabetic cardiomyopathy are endothelial dysfunction, deterioration of microcirculation, development of myocardial fibrosis and metabolic disorders, such as high free fatty acids levels, calcium homeostasis imbalance and lacking in carnitine [4,5].

Echocardiography is a time-sparing and cost-effective method that provides accurate and reproducible diagnostic and prognostic information in patients with DM [6]. The application of two-dimensional, doppler and speckle tracking echocardiography, help us to thoroughly interrogate cardiac function in diabetics [7], while stress echocardiography and evaluation of coronary flow reserve provide incremental prognostic value [8,9].

## 2. Diastolic Dysfunction

Recent data show that deterioration of LV diastolic function is quite usual among patients with DM, regardless of clinical status [10]. This means that asymptomatic diabetics manifest subclinical myocardial dysfunction and are prone to the development of clinical syndrome of heart failure, which has adverse impact on their prognosis. Consequently, echocardiographic assessment in a timely manner is critical for the optimal management of patients with DM in order to detect such subclinical abnormalities. Important studies referring to diastolic dysfunction in diabetics are mentioned in Table 1.

Echocardiographic abnormalities are present in diabetics, despite the fact that symptoms or clinical characteristics do not mandate echocardiographic assessment. Joergensen et al. studied 1030 patients with DM type 2 and revealed remarkable abnormalities through echocardiographic assessment. More precisely, in half of these patients, researchers unveiled diastolic dysfunction with normal or elevated end-diastolic pressure, left atrial dilatation or LV hypertrophy. These findings were not associated with patient’s clinical profile and, as a result, clinical details could not discriminate subjects with deteriorated diastolic function, as assessed by echocardiography. Thus, it is implied that echocardiographic assessment should be an essential screening tool for diabetic patients, regardless of their clinical characteristics [11].

Moreover, diastolic dysfunction can occur even in young individuals in the short term after DM manifestation. According to recent research, deterioration of LV diastolic properties is more common in diabetics compared to control subjects. In fact, they noticed that the higher the HbA1c and the longer the span of diabetes, the greater the probability that markers of diastolic function will be impaired [12]. Older studies have also reported that diastolic impairment in diabetic patients is of high prevalence [13].

Furthermore, it is claimed that LV diastolic function abnormality may be detected by echocardiography even in the first five years after the diagnosis of diabetes. According to previous studies referring to diabetics, diastolic dysfunction is associated with defective glucose regulation. Metabolic parameters, such as high glucose plasma levels, might also enhance oxidative stress, intoxicate the myocardial cell, and promote the development of diabetic cardiomyopathy [14]. Similarly, Celentano et al. suggested that the propensity for developing diastolic dysfunction is proportionate to the fasting glucose bloodstream levels [15], while another research group underlined the association between uncontrolled DM, as defined by high HbA1c, and the danger of suffering from heart failure [16].

Another research group assessed 1134 patients with diabetes heart failure via preserved ejection fraction. Diabetics displayed bigger volumes of cardiac chambers and increased mass of left ventricle. Moreover, markers of diastolic function, such as trans-mitral E/A ratio, Isovolumic Relaxation Time (IVRT), Deceleration Time (DT), pulmonary vein doppler, and e’ derived from Tissue Doppler unveiled deteriorated ventricular compliance with elevated left ventricular end-diastolic pressure. These findings were in concordance with increased levels of N-terminal prohormone of brain natriuretic peptide (NT-proBNP), which were detected in patients with diabetes mellitus, participating in an I-Preserve trial [17]. 

Moreover, by studying diabetics with coronary artery disease with Doppler echocardiography and intracoronary ultrasound in order to qualify left ventricular diastolic properties, to assess epicardial plaque burden and microcirculation and to determine coronary flow reserve (CFR), it was concluded that there is a relationship between diastolic function deterioration and devastation of microcirculation [1].

Apart from patients with DM type 2, in patients with DM type 1, despite being younger and manifesting less comorbidities, echocardiographic evaluation plays a critical role in risk stratification and has an incremental value on clinical risk scores. In a recent trial, 1093 individuals with DM type 1 were studied during a period of 7.5 years. Echocardiographic parameters, such as Left Ventricular Ejection Fraction (LVEF), impaired global longitudinal strain (GLS) and E/e’ ratio were the main determinants of prognosis. Consequently, assessment of these markers improved the early detection of individuals in jeopardy beyond conventional clinical risk factors [18].

Moreover, left atrial evaluation is feasible by means of speckle tracking echocardiography. In accordance with a recent study, left atrial enlargement in patients with DM is not related to the presence of hypertension or diastolic dysfunction. Actually, left atrial dilatation is combined with deteriorated atrial longitudinal strain. More precisely, DM is likely to affect left atrial function and this abnormality leads gradually to left atrial enlargement [19]. 

In summary, echocardiography is likely to detect diastolic abnormality and subtle myocardial dysfunction in asymptomatic diabetics before symptoms occur. In such cases, medical treatment should be optimized, in order to prevent further devastation of heart function.

## 3. Global Longitudinal Strain (GLS)

Diabetic cardiomyopathy is characterized by myocyte hypertrophy and fibrosis development, which may affect myocardial contractility [20].

Speckle tracking echocardiography is a contemporary tool that analyzes myocardial deformation. It is feasible to detect subtle myocardial impairment in diabetics, before symptoms arise and before conventional echocardiography detects malfunction. Longitudinal deformation of the left ventricle is the most scrutinized mode, but also radial and circumferential deformation have been thoroughly studied [21]. Important studies referring to myocardial deformation in diabetics are mentioned in Table 2.

Global longitudinal strain (GLS), assessed by speckle tracking echocardiography (STE), was used to detect preclinical diabetic cardiomyopathy. GLS was impaired in patients with DM1 compared to controls. The recruited subjects had no history of cardiovascular disease and no additive risk factors. Nevertheless, they manifested subtle myocardial deterioration, which could not be detected by conventional markers of cardiac function, such as ejection fraction or tissue doppler systolic velocity [22].

Similarly, the aim of another trial was to see if patients with DM type 1 develop preclinical, subtle biventricular functional deterioration. They examined asymptomatic subjects without either documented diabetic complications or known cardiovascular ramifications. After investigating thoroughly left and right ventricular longitudinal mechanics, they claimed that longitudinal deformation is abnormal in subjects with DM type 1. Additionally, they reported that the deformation impairment was associated with how long the disease lasted and how well the disease was controlled [23].

Asymptomatic subjects with DM type 1 were studied thoroughly with exercise echocardiography. Parameters assessed were longitudinal and circumferential strain and strain rate, by STE, at rest and at peak exercise. Subjects were compared with healthy controls. Despite that asymptomatic subjects with DM type 1 and a short duration of the disease have good adaptation to exercise, as was implied by rising GLS and Global Circumferential Strain (GCS), poor compliance with treatment and improper diabetes regulation enhanced myocardial dysfunction [24].

It is also claimed that obesity is an additive hazard factor for devastating myocardium in diabetics. After studying diabetic patients with a free history of cardiovascular disease and with preserved ejection fraction, researchers concluded that increased body weight induced subclinical alteration in longitudinal deformation, precisely detected by STE. As a result, we notice that while DM causes a subtle and global deterioration in cardiac function before symptoms occur, obesity further hampers proper systolic and diastolic function [25]. 

Moreover, researchers hypothesized that Galectin-3, an established biomarker for monitoring patients with heart failure with reduced ejection fraction, might be in concordance with GLS deterioration and might identify subtle abnormality in left ventricular systolic properties of diabetics with preserved ejection fraction. Actually, they searched for an association between a potent rise in galectin levels and development of subclinical diabetic cardiomyopathy. According to their results, galectin-3 was increased in the study population, consisting of diabetics with preserved ejection fractions, and indeed patients with impaired longitudinal deformation, assessed by Speckle Tracking Echocardiography, had higher serum levels of galectin-3. Rising galectin-3 and reduced GLS in diabetics with preserved ejection fraction imply left ventricular remodeling and development of diabetic cardiomyopathy [26].

On the other hand, another research group aimed to investigate whether subtle myocardial dysfunction, assessed by GLS, is a trait of uncomplicated DM or whether it is a complication of diabetes that occurs in later stages of the disease, when multisystem complications have been induced. With that purpose they compared normal controls, uncomplicated subjects with DM type 1 and subjects with DM type 1 and albuminuria. All subjects were free of cardiovascular disease. In concordance with previous trials, subtle abnormality in myocardial contractility, detected by deteriorated longitudinal deformation, was noticed in diabetics. Nevertheless, it was further claimed that the occurrence of diabetic cardiomyopathy was related to the detection of systematic diabetic complications, such as albuminuria, and not just with subclinical deterioration in GLS [27].

So far, evidence supports that STE provides a diagnostic tool in order for preclinical myocardial dysfunction to be detected. The prognostic impact of this information was further validated by studying 247 patients with DM type 2 without a history of cardiovascular events. In this group of patients, abnormal GLS had additive value over conventional risk factors in hazard assessment [28].

Beyond risk assessment, response to treatment can efficiently be monitored through estimating longitudinal deformation. Subjects with DM type 2 were upgraded to optimal medical treatment with regard to conventional risk factors and in accordance with contemporary guidelines. GLS, and early diastolic velocity by tissue doppler (e’) were measured, in order for left ventricular systolic and diastolic properties respectively to be evaluated. Optimal medical treatment for 12 months contributed to amelioration of GLS and e’, thus myocardial function was protected by the intensification of treatment [29].

Furthermore, a study group tried to determine the echocardiographic features of diabetes in patients without a history of cardiovascular disease. Study population included healthy controls and asymptomatic, uncomplicated, diabetic subjects with preserved ejection fraction, normal left ventricular mass index, normal diastolic function parameters and BNP in normal range. They investigated longitudinal, radial, and circumferential deformation globally and afterwards separately for endocardium, midcardium and epicardium. They concluded that uncomplicated diabetes, in patients without comorbidities and without additional risk factors, formulates a specific pattern in the left ventricle, assessed by STE, which is characterized by impaired GLS and deteriorated subendocardial radial deformation [30].

In a similar manner, in 52 patients with DM type 1, investigators performed layer analysis of left ventricular walls by STE. While longitudinal strain was affected globally in the left ventricle, circumferential strain was impaired segmentally. The pattern included impaired circumferential strain at the external layers of the ventricular base and was more overt as the disease was in progress for a longer time period [31].

Moreover, another study group recruited patients with heart failure and preserved ejection fraction and examined diastolic function parameters and left atrial deformation. The aim of the study was to investigate whether presence of diabetes has an incremental role in affecting these parameters. Indeed, in diabetics left atrial strain was significantly impaired in comparison with the other patients and this implies that left atrial function is much more deteriorated in diabetics. As a result, left atrial deformation has incremental predictive value in patients suffering from heart failure with preserved ejection fraction [32].

With regard to another research group, left atrial volume does not accurately reflect atrial function. To support this hypothesis, they examined subjects with diabetes, subjects with hypertension and subjects with both diabetes and hypertension. According to their results, left atrial strain was abnormal in these patients, despite the fact that left atrial dimensions were in normal range. Additionally, they showed that deterioration of left atrial performance was more severe when subjects suffered concomitantly from both risk factors. Thus, investigation of left atrial deformation may reveal subtle atrial dysfunction early in the process of a disease, while left atrial volume remains normal [33].

Furthermore, apart from DM, it seems that acute hyperglycemia may also adversely affect left ventricular performance. More specifically, left ventricular deformation was impaired in asymptomatic subjects with diabetes after an episode of acute hyperglycemia. Circumferential and longitudinal parameters, derived from STE, were mainly detected to be affected. An assessment after three months showed that affected parameters were still deteriorated [34].

Acute hyperglycemia postprandially also seems to impair cardiac function. After studying first degree relatives of diabetics type 2 with normal oral glucose tolerance test (OGTT) and patients with abnormal OGTT, it was concluded that the OGTT had an impact on deformation parameters. Investigators claimed that both global and subendocardial longitudinal strain were deteriorated after the test, while left ventricular twisting and untwisting became more intense. According to the authors, this is explained by the fact that hyperglycemia deteriorates endocardial deformation and has neutral effect on epicardial deformation which gains a dominant role [35].

Last but not least, according to another study, speckle tracking echocardiography could be used as a method to evaluate treatment’s effect in cardiac function. Actually, researchers recruited patients with DM type 2, with or without coronary artery disease and treated them according to contemporary guidelines. Subjects formed four groups with regard to treatment with insulin, glucagon-like peptide-1 receptor agonists (GLP-1RA), sodium-glucose cotransporter-2 inhibitors (SGLT-2i) or GLP-1RA and SGLT-2i combination. After 12 months, GLP-1RA and SGLT-2i improved markers such as effective cardiac work and myocardial deformation compared with insulin treatment. The combined therapy (GLP-1RA and SGLT-2i) had even better effects on studied parameters [36,37].

In summary, speckle tracking echocardiography might detect subtle impairment in myocardial function, which has an incremental value in risk stratification and in response to therapy of diabetic patients with or without coronary artery disease.

## 4. Stress Echo

Stress echocardiography is a validated technique for the detection and risk stratification of coronary artery disease. By adding wall motion assessment during exercise and coronary flow reserve to clinical features, electrocardiographic changes and blood pressure adaptation to exercise, the efficiency of exercise to diagnose CAD is remarkably enhanced. Stress echocardiography is an affordable, cost-effective, radiation-free method and its detective and predictive capacity are similar to other noninvasive imaging techniques, such as magnetic resonance imaging and nuclear cardiology. Important studies referring to stress echocardiography in diabetics are mentioned in Table 3.

In fact, stress echo is safe for diabetic patients. Detection of segmental wall motion abnormalities during the test is a risk factor for the development of major adverse cardiovascular events. A normal or a pathologic response to stress echo obtains a different meaning with regard to pre-test probability. Despite normal response to stress, patients with reduced ejection fraction at rest or diabetics are still at higher risk compared with subjects with normal ejection fraction or non-diabetics, respectively. The diabetics in jeopardy for adverse cardiovascular events, regardless of a quite good response to stress test, are mainly older, with impaired ejection fraction, and unable to exercise efficiently. Moreover, subjects with DM, in whom a stress echo detected ischemia, are at bigger peril for developing major adverse cardiac events, compared to subjects without DM. Reduced ejection fraction at rest and an increased number of ischemic segments further increase cardiac risk [38]. 

As a result, stress echo is an efficient method for risk stratification that adds significant information. Nevertheless, it is proven that stress echo is not a screening tool for the general population and consequently assessment without an indication does not provide any predictive evidence [39]. 

This was also the aim of a recent study to assess whether compliance with pharmaceutical stress echo indications further enhances the impact of the method. The result was more or less obvious, as the method was proved to be effective when practitioners adhered to established indications and official guidelines. Diabetes per se is not an indication for performing a stress echo and therefore the method has no incremental predictive role in diabetics unless specific criteria are met. Therefore, clinicians should not refer inappropriate patients for stress echo evaluation even if they are diabetics [40]. This is also supported by the European Society of Cardiology (ESC), which reports that, although noninvasive methods might be used with regard to clinical discretion, these should not be used as a screening method for coronary artery disease in the general population or in special populations, such as diabetics. Nevertheless, stress echo might be performed in subjects without symptoms but, concomitantly, at great peril for developing major adverse cardiovascular events. These patients actually manifest peripheral artery disease, chronic kidney disease, high calcium score or proteinuria [41].

Screening all diabetics for coronary artery disease is improper, since it has been shown that a low rate of 5–10% of asymptomatic individuals with diabetes have significant lesions in coronary arteries. Only patients with specific traits may be eligible for being screened with stress echo, such as those needing pancreas or renal transplantation [42] or undergoing severe noncardiac invasion [43]. On the contrary, symptomatic subjects with DM, with typical symptoms or not, and individuals with established, or at high risk for, cardiovascular disease are supposed to benefit from screening [38]. This should be emphasized and is also supported by emerging data from more studies. To be more specific, investigators, after assessing subjects that did not report clinical defects, but had already manifested diabetic microvascular or macrovascular ramifications, noticed that 25% of the study participants had impaired wall motion score index and coronary flow reserve. This obviously had a negative impact on prognosis for these individuals [44].

Moreover, a research group tried to examine the role of stress echocardiography in determining prognosis of subjects with DM. Indeed, they reported that, after evaluating clinical features, baseline echo and stress echo information, they created a predictive model. By using this, they managed to distinguish 2349 individuals with DM in three different levels of risk for adverse cardiovascular events [45].

Similarly, another study claimed that stress echocardiography is an elaborative method to evaluate potency for adverse cardiac outcome in diabetics with speculated coronary artery disease. Actually, they assessed the predictive value of dobutamine stress echo in 325 such individuals during a period of 34 months. Despite the fact that univariate analysis showed high cardiac mortality after a positive response in the stress test, finally only older age and peak ejection fraction <40% were independently associated with cardiac death. Furthermore, when the study subjects were distinguished according to baseline EF in two groups, it was shown that positive response in stress test and EF at high level of stress <40% were independently correlated with cardiac mortality [46].

Due to the fact that CAD is the primary reason of cardiac mortality in subjects with DM, scientists search for methods to further determine cardiovascular risk. Thus, 193 diabetics were recruited and studied for two and a half years. Diabetics with findings of ischemia on Exercise Echocardiography (EE) had worse cardiac outcomes compared to general population. Thereafter, EE could indeed be a tool for the prediction of adverse outcomes in diabetics with cardiovascular disease [47].

While ischemia at stress echo is an important indicator of mortality in both groups of patients with and without diabetes, it seems that antianginal medication alters the negative predictive value of the method only in subjects without DM but not in subjects with DM [48].

According to the results of a recent study, stress echo seems to be important for risk assessment of individuals with DM. A negative for ischemia stress test is associated with good prognosis, regardless of the method used. However, the risk in diabetics is definitely higher. Nevertheless, detection of ischemia in a stress test is independently related with adverse events and the grade of peril is associated with the extent of ischemia detected [49]. 

It has been shown that stress echocardiography in diabetics is less sensitive than perfusion imaging due to concomitant diabetic cardiomyopathy. On the other hand, it is more specific compared to myocardial perfusion imaging. This may be explained by the fact that in these patients both microcirculation and coronary vessels are affected. In comparison with normal subjects, in patients with diabetes a normal response to stress is followed by a worse outcome [50].

On the other hand, a recent study reported that, in diabetic individuals who are unlikely to complete an exercise test, the pharmaceutic test has a reduced capability of foreseeing future adverse events. It was also suggested that there is a period of up to seven years after the test in which optimal risk stratification is provided [51]. 

Finally, according to a trial which compared 44 diabetics with 35 healthy nondiabetics, it was claimed that dobutamine stress echo might detect subtle alterations in left ventricular deformation properties, which were not overt before the stress test. In particular, left ventricular stimulation by low dose dobutamine led to deterioration of left ventricular longitudinal deformation, circumferential strain, rotation and twist [52].

In summary, it is obvious that when clinicians comply with the appropriateness criteria of stress echocardiography, the method is likely to contribute to the optimal management of individuals with diabetes. Actually, stress echocardiography can unveil the existence of CAD and may evaluate the risk for major adverse cardiovascular events in individuals with DM. 

## 5. Coronary Flow Reserve (CFR)

Echocardiography, apart from unveiling preclinical myocardial dysfunction in subjects with DM, might further contribute to risk stratification in this group of patients. Innovative and state-of-the art methods, such as Coronary Flow Reserve Assessment by Doppler Echocardiography, may detect endothelial dysfunction and deterioration of both epicardial and microvascular circulation that are proportional with the severity of diabetes. Important studies referring to coronary flow reserve in diabetics are mentioned in Table 4.

The value of CFR in risk assessment of asymptomatic individuals with DM is crucial. Researchers investigated asymptomatic subjects with DM type 2 without established CAD and validated the prognostic role of CFR in this population. After excluding subjects with CFR < 2, because of increased probability of having a severe coronary lesion, they reported that CFR ≥ 2.5 was related to fewer adverse cardiovascular events. On the other hand, patients with 2 < CFR < 2.5 had a poorer prognosis. Thus, CFR helps us in achieving a more precise risk assessment of patients with DM [53]. 

Other investigators studied patients admitted to hospital due to chest pain and with speculated coronary artery disease. Among them, diabetic patients had more significant aortic atherosclerosis and lower CFR, while significant CAD was more common in diabetics. CFR and diabetes were independent predictors of cardiovascular survival. Among subjects recruited, diabetics with decreased CFR, which implied devastated microcirculation, were at bigger peril for developing cardiovascular complications [54].

Another group of researchers attempted to explore coronary microcirculation of patients with insulin resistance in comparison with diabetic patients and normal population. While CFR was reduced in patients with DM, patients with insulin resistance (nondiabetics officially) and normal controls had similar CFR. With regard to this study, it is claimed that microcirculatory dysfunction is detected after the outbreak of DM [55].

On the contrary, it was reported by another research group that first-degree relatives of diabetics with normal OGTT and subjects with abnormal OGTT had comparable resistance to insulin, similarly defective arterial elastic properties and diminished CFR compared to controls. According to these results, it was concluded that resistance to insulin is a contributing factor to the development of subtle deterioration of vascular, microvascular and myocardial function [56].

Moreover, researchers tried to evaluate whether CFR decline in diabetics is associated with insulin resistance or affected by acute hyperglycemia. On that purpose, they investigated individuals with DM2 and controls. Despite that CFR declined in diabetics compared with controls, it was reported that proper glucose regulation, as assessed by fasting glucose and HbA1c levels, was correlated with less diminished CFR. Insulin resistance was not correlated with CFR. Thus, in type 2 diabetics, glucose regulation is most likely to be associated with the decline in CFR [57].

Finally, questions had been raised as to whether CFR is similar among diabetic patients with complications and without complications. As a result, researchers studied diabetic patients with or without diabetic retinopathy. It was concluded that coronary flow reserve was remarkably diminished in diabetics complicated with diabetic retinopathy. Despite that CFR is generally decreased in diabetics, the presence of a complication such as retinopathy, which implies a more advanced stage of diabetes, is also associated with a more intense deterioration of microcirculation. Consequently, the more advanced the development of retinopathy, the more exaggerated the impairment in microvascular function, as assessed by coronary flow reserve [58].

In summary, CFR plays a major role in the diagnosis of microvascular dysfunction in diabetics. Additionally, when CFR is combined with stress echocardiography, it has incremental role in the diagnosis of coronary artery disease and risk stratification in patients with DM.

## 6. Clinical Implications

Echocardiography is an efficient tool for the management of diabetics [59]. In fact, patients with DM2 should essentially be assessed by echocardiography at the time of the diagnosis. Afterwards, some key factors, such as presence of coronary artery disease or hypertension, poor diabetes control, disease duration and clinical status, may determine how often the examination should be repeated [60]. For subjects with DM1 echocardiography is also useful. Despite manifesting less comorbidities than individuals with DM2, subclinical myocardial function often occurs and optimal management necessitate echocardiographic assessment. Furthermore, stress echo should be performed in symptomatic patients with moderate probability for coronary artery disease detection, while asymptomatic subjects at high risk for developing major adverse cardiovascular events, such as those with peripheral artery disease, chronic kidney disease, high calcium score or proteinuria, might also undergo the examination. A negative exam implies that stress echo might not be repeated for almost five years unless clinical deterioration occurs [41].

## 7. Conclusions

Proper assessment of diabetic patients is critical, in order to hamper the development of vascular and microvascular complications. Echocardiography is a feasible and safe method that provides diagnostic and predictive information in individuals with diabetes mellitus. A diversity of applications, such as Doppler echocardiography, Speckle Tracking Echocardiography and Stress Echocardiography, could be useful for the management of diabetics and the optimization of medical treatment. Contemporary standard of care in diabetics should include echocardiography for clinical assessment.

## Figures and Tables

**Table 1:** Studies referring to diastolic function in diabetics.

First Author/Study Name	Study Population	Features Studied	Endpoints
Joergensen, et al. (Diab Vasc Dis Res. 2016)	1030 DM2	Clinical, electrocardiographic, echocardiographic (LVMI, LVEF, LAVi, E/e’, TAPSE), biochemical parameters (HbA1c, lipid profile, creatinine, urine albumin).	Echocardiographic abnormalities are common in DM2, independently of clinical biochemical characteristics.
Ashour, et al. (J Heart Cardiovasc Res. 2018)	86 DM vs 65 age and sex matched controls	Clinical, echocardiographic (LVESD, LVEDD, LVEF, LAVI, E/A ratio, E/e’, TR Velocity) and biochemical parameters (HbA1c).	Diastolic dysfunction can occur even in young subjects in the short term after DM manifestation.
Romano, et al. (Cardiovasc Diabetol. 2010)	127 DM2	Clinical, echocardiographic (LVESD, LVEDD, LVEF, LVMI, E/A ratio, DT, S/D) and biochemical parameters (HbA1c, creatinine, urine albumin, BNP).	BNP may discriminate asymptomatic patients manifesting impaired diastolic function among uncontrolled diabetics.
Celentano, et al. (Am J Cardiol. 1995)	25 normoglycemia 15 IGT 24 NIDDM.	Clinical, echocardiographic (LVESD, LVEDD, LVEF, LVFS, LVMI, E/A ratio, DRWT) and biochemical parameters (fasting Glucose, OGTT).	Both subjects with insulin resistance and diabetics are prone to develop abnormalities of left ventricular diastolic properties.
Kristensen, et al. (Circulation. 2017) (i-Preserve trial)	1134 DM out of 4128 patients of i-Preserve trial	Clinical, electrocardiographic, echocardiographic (LV volumes LVEF, LVFS, LVMI, LAVI, E/A ratio, E/e’, DRWT, DT, IVRT, TR Velocity) and NT-proBNP.	Among patients with HFPEF, in diabetics we detect the most extensive echocardiographic and clinical deterioration, increased NT pro-BNP and the worst outcome.
Escaned, et al. (Rev Esp Cardiol. 2009)	13 DM and CAD.	Echocardiographic (LVESD, LVEDD, LVEF, LVMI, E/A ratio, E/e’, DRWT, DT, IVRT), intracoronary echocardiographic (vessel diameter & area, luminal diameter & area, plaque volume) and hemodynamic parameters (intracoronary pressure & flow velocity, CFR).	In subjects with DM & coronary atherosclerosis, LV diastolic dysfunction is related to alterations in the coronary microcirculation.
Jensen, et al. (Diabetologia. 2019) (Thousand & 1 Study)	1093 DM1	Clinical, echocardiographic (LVESD, LVEDD, LVEF, E/A ratio, E/e’, GLS).	Echocardiography has an incremental role in hazard assessment of patients with DM type 1 and without a history of cardiovascular disease.
Kadappu, et al. (Eur Heart J Cardiovasc Imaging. 2012)	73 DM vs age- and gender-matched controls	Clinical and echocardiographic parameters (LV volume, LVEF, LVMI, LAVI, E/A ratio, E/e’, DT, IVRT, LA global strain and strain rate, LA EF).	In subjects with DM, LA dilatation is the result of LA dysfunction, as detected by LA deformation study (speckle tracking echocardiography (STE)), regardless of the coexistence of hypertension or diastolic abnormality.

CAD: Coronary Artery Disease, CFR; Coronary Flow Reserve, CV: Cardiovascular, DRWR: Diastolic Relative Wall Thickness, DM: Diabetes Mellitus, DT: Deceleration Time, EDV: End Diastolic Volume, ESV: End Systolic Volume, EF: Ejection Fraction, FS: Fractional Shortening, GLS: Global Longitudinal Strain, HbA1c: Hemoglobin A1c, IVRT: Isovolumic Relaxation Time, IGT: Impaired Glucose Tolerance, LA: Left Atrial, LAVI: Left Atrial Volume Index, LV: Left Ventricular, LVDF: Left Ventricular Diastolic Function, LVESD: Left Ventricular End Systolic Dimension, LVEDD: Left Ventricular End Diastolic Dimension, LVMI: Left Ventricular Mass Index, NIDDM: Non-Insulin dependent Diabetes Mellitus, NT-proBNP: N-terminal prohormone of brain natriuretic peptide, OGTT: Oral Glucose Tolerance Test, S/D: Pulmonary Venous Systolic & Diastolic Flow Velocity, TAPSE: Tricuspid Annular Plane Systolic Excursion, TR: Tricuspid Regurgitation.

**Table 2:** Studies referring to GLS in diabetics.

First Author/Study Name	Study Population	Features Studied	Endpoints
Fang, et al. (J Am Coll Cardiol. 2003)	48 DM, 45 LVH, 45 DM and LVH 48 normal controls.	Clinical and echocardiographic (LV dimensions and Volumes, LVEF, LVMI, TDI measured Strain and Strain Rate).	Longitudinal strain and strain rate are impaired before the reduction of LVEF in DM and is independent of LVH.
Ringle, et al. (Echo Res Pract. 2017)	66 DM1 26 controls.	Clinical, 2D echocardiographic (LVESD, LVESV, LVEDD, LVEDV, LVEF, LVMI, LAVI, E/A ratio, E/e’, DT, GLS, GCS, GRS, GLSR, GCSR, GRSR, SRE, E/SRE by STE) and 3D echocardiographic (LVESD, LVESV, LVEDD, LVEDV, LVEF, GLS, Torsion, Twist) parameters.	Myocardial deformation study by STE is able to discriminate subtle alteration of left ventricular function in subjects with DM type 1.
Zairi, et al. (Indian Heart J. 2019)	52 DM1 52 matched controls	Clinical and 2D echocardiographic parameters (LVEF, LVMI, LAVI, RVEDD, RVFWT, FAC, RAV, TAPSE, E/A, TDI parameters, E/e’, LV GLS, RV FWLS by STE).	LV GLS and RV FWLS are decreased in young individuals with DM1 compared to controls. The degree of impairment is related to glucose regulation duration and the span of the disease.
Hensel, et al. (J Diabetes Res. 2016)	40 DM1 44 controls.	Clinical, 2D echocardiographic parameters (LVESD, LVESV, LVEDD, LVEDV, LVEF, IVSEDD, LVPWEDD, DRWT, LV FS, LVMI, LAVI, E/A ratio, DT, IVRT, TDI parameters, E/e’, SVi, GLS and GCS by STE during rest and exercise).	Despite that asymptomatic DM1 have increased GLS & GCS at rest and at peak exercise in the short term after diagnosis, suboptimal glucose regulation leads to myocardial dysfunction.
Conte, et al. (J Cardiovasc Echogr. 2013)	71 DM 24 healthy controls.	Clinical and 2D echocardiographic (LVESD, LVESV, LVEDD, LVEDV, LVEF, LVMI, LAVI, E/A ratio, DT, SVi, TDI parameters, E/e’, DT, GLS, GCS, GRS, GLSR) parameters.	In uncomplicated DM, despite normal EF&TDI, GLS by STE is found impaired. Obesity further contributes to LV deformation deterioration.
Flores-Ramírez, et al. (Arch Cardiol Mex. 2017)	90 DM 31 controls.	Clinical, 2D echocardiographic parameters (LVESD, LVESV, LVEDD, LVEDV, LVEF, GLS by STE) and galectin.	In individuals with DM and mrEF, galectin is high and GLS is declined,
Jensen, et al. (JACC Cardiovasc Imaging. 2015)	1065 DM1 198 healthy controls.	Clinical, 2D echocardiographic parameters (LVESD, LVESV, LVEDD, LVEDV, LVEF, LVMI, LAVI, E/A ratio, TDI parameters, E/e’, DT, GLS by STE) and urine albumin.	GLS was reduced in DM1 compared with controls and particularly in DM1 patients with albuminuria.
Liu, et al. (Cardiovasc Diabetol. 2016)	247 DM2	Clinical and 2D echocardiographic parameters (LVESD, LVESV, LVEDD, LVEDV, LVEF, IVSEDD, LVPWEDD, DRWT. LVMI, LAVI, E/A ratio, DT, TDI parameters, E/e’, GLS by STE).	Myocardial deformation is abnormal in subjects with DM type2 and this has an impact on the risk assessment.
Leung, et al. (Circ Cardiovasc Imaging. 2016)	105 DM2 and poor glycemic control.	Clinical and 2D echocardiographic parameters (LVESV, LVEDV, LVEF, IVSEDD, LVPWEDD, LVMI, LAVI, E/A ratio, DT, TDI parameters, E/e’, GLS by STE).	Myocardial function was ameliorated after optimal diabetes regulation for a period of 12 months.
Enomoto, et al. (Circ J. 2015)	104 poorly controlled DM2, 24 matched controls.	Clinical, 2D echocardiographic parameters (LVEF, IVSEDD, LVPWEDD, DRWT, LVMI, LAVI, E/A ratio, DT, TDI parameters, E/e’, S/D, GCS & GRS & GLS by STE) and NT-proBNP.	In poorly controlled type 2 diabetics deterioration of GLS and subendocardial radian strain are the echocardiographic parameters which are initially affected.
Iso, et al. (Circ J. 2019)	52 patients with DM1 were divided into 3 age groups.	Clinical and 2D echocardiographic parameters [LVEF, LVSEDD, LVPWEDD, LVMI, LAVI, E/A ratio, DT, TDI parameters, E/e’, LV layer-specific strain analysis (CS basal/papillary/apical, LS) by STE].	In teenagers with DM1, a specific pattern of longitudinal and circumferential strain deterioration was detected and this was related to the disease duration and the presence of LV hypertrophy.
Georgievska-Ismail, et al. (Diab Vasc Dis Res. 2016)	218 HFPEF divided according to the presence of DM.	Clinical and 2D echocardiographic parameters (LVESD, LVESV, LVEDD, LVEDV, LVEF, IVSEDD, LVPWEDD, DRWT, LVMI, LAVI, LA EF, E/A ratio, DT, IVRT, TDI parameters, E/e’, S/D, LA peak longitudinal & contraction strain by STE).	2D STE may unveil deterioration of left atrial function in patients with HFPEF and DM.
Mondillo, et al. (J Am Soc Echocardiogr. 2011)	155 patients with hypertension or DM with LA volume indexes < 28 mL/m2 and 36 age-matched controls.	Clinical and 2D echocardiographic (LVESV, LVEDD, LVEF, IVSEDD, LVPWEDD, DRWT, LVMI, LAVI during different phases of cardiac cycle, LA EF, E/A ratio, DT, TDI parameters, E/e’, global LA longitudinal strain and strain rate by STE) parameters.	Investigation of left atrial deformation may reveal subtle atrial dysfunction early in the process of DM and HT, despite left atrial volume remaining normal.
Bogdanović, et al. (Cardiovasc Diabetol. 2019)	67 DM with acute hyperglycemia, 20 DM with optimal metabolic control, 20 healthy controls.	Clinical, 2D echocardiographic [LVESV, LVEDV, LVEF, IVSEDD, LVPWEDD, LAVI, E/A ratio, DT, E/e’, LV layer-specific strain analysis (CS basal/papillary/apical, LS by STE)] and biochemical (HbA1c, fasting glucose, Troponine, NT-proBNP) parameters.	Acute hyperglycemia deteriorates left ventricular performance in individuals with DM, as assessed by STE, and this deterioration does not inverse after three months of proper glucose regulation.
Ikonomidis, et al. (Int J Cardiol. 2017)	100 subjects 40 first degree relatives of DM, normal OGTT 20 normal OGTT, no family history of diabetes 40 abnormal OGTT.	Clinical, 2D echocardiographic [GLS, LV layer-specific strain analysis (LS endo/mid/epicardial), Twisting/Untwisting by STE], vascular (PBR) and biochemical (OGTT, Insulin sensitivity Index) parameters.	First-degree relatives of diabetics and subjects with abnormal OGTT have impaired glycocalyx, deteriorated LV longitudinal deformation and twist.
Ikonomidis, et al. (J Am Heart Assoc. 2020)	160 patients DM2	Clinical, 2D echocardiographic (GLS, GLSR, GCS, GRS, GWI, GCW, GWW, pTw, pUtwVel by STE), and vascular (PBR of sublingual arterial microvessels, PWV).	Adding GLP-1RA or SGLT-2i in subjects with DM2 ameliorated vascular and myocardial function compared with insulin. Their combination further improved vascular and myocardial function.
Lambadiari, et al. (J Clin Med. 2019)	100 poorly controlled DM2	Clinical, 2D echocardiographic (LVEF, GLS, GLSR S, GLSR E, GLSR A, LV twisting-untwisting LAVI, E/A, E/e’ by STE), vascular (PBR of sublingual arterial micro-vessels, PWV, AI, FMD) and biochemical (oxidative stress markers, HbA1c, lipid profile, fasting glucose, creatinine) parameters.	Proper glucose regulation with the addition of GLP-1RA and SGLT-2 reduces oxidative stress and enhances optimal myocardial and vascular function of DM2 patients.

A: peak trans-mitral late diastolic velocity, AI: Augmentation Index, BP: blood pressure, BMI: Body Mass Index, CAD: Coronary Artery Disease, CFR; Coronary Flow Reserve, DM: Diabetes Mellitus, E: peak trans-mitral early diastolic velocity, E′: peak earl mitral annular velocity, FMD: percentage difference of Flow-Mediated Dilatation, GCW: Global Constructive Work, GWI: Global Work Index, GWW: Global Wasted Work, IVS-EDD: Interventricular Septal End-diastolic Dimension, FAC: Fractional Area Change, FWLS: Free Wall Longitudinal Strain, GCS: Global Circumferential Strain, GFR: Glomerular Filtration Rate, GLP-1RA: Glucagon like peptide Receptor Agonist, GLS: Global Longitudinal Strain, GLSR: Global Longitudinal Strain Rate, GRS: Global Radial Strain, HbA1C: hemoglobin glycosylated, HFPEF: Heart Failure with Preserved Ejection Fraction, LA: Left Atrium, LAVI: Left Atrial Volume Index, LV: Left Ventricular, LVEDD: Left Ventricular End-Diastolic Dimension, LVEDV: Left Ventricular End-Diastolic Volume, LVESD: Left Ventricular End-Systolic Dimension, LVESV: Left Ventricular End-Systolic Volume, LVMI: Left Ventricular Mass indexed to estimated body surface area, LVPW-EDD: Left Ventricular Posterior Wall End-diastolic Dimension, LVEF: Left Ventricular Ejection Fraction, LVH: Left Ventricular Hypertrophy, mrEF: mildly reduced Ejection Fraction, NIDDM: Non-Insulin dependent Diabetes Mellitus, PBR: Perfused Boundary Region, pTw: peak twisting, pUtwVel: peak untwisting velocity, PWV: pulse wave velocity, RV: Right Ventricular, RAV: Right Atrial Volume, RV-EDD: Right Ventricular End-diastolic Diameter, RV Free Wall: Right Ventricular Free Wall, RV FWLS: Right Ventricular Free-Wall Global Longitudinal Strain, S/D: Pulmonary Venous Systolic & Diastolic Flow Velocity, SGLT-2i: Sodium-Glucose Cotransporter inhibitor, STE: speckle tracking echocardiography, SRE: strain rate at early diastole, SVi: Stroke Volume Index, TAPSE: tricuspid annular plane systolic excursion, TDI: Tissue Doppler Imaging.

**Table 3:** Studies referring to stress echo in diabetics.

First Author/Study Name	Study Population	Features Studied	Endpoints
Aggeli, et al. (Int J Cardiol. 2016)	574 DM	Clinical and echocardiographic (LVEF and RWMA) parameters.	DSE is a strong prognostic predictor for MACE (all-cause mortality, cardiovascular mortality, late revascularization (performed >3 months after DSE) and hospitalizations) in diabetics with clinical indication of stress test.
Bates, et al. (Circ Cardiovasc Imaging. 2015)	53 insulin-dependent DM	Clinical and echocardiographic (LVESD, LVEDD, LVEF, IVSEDD, LVPWEDD, LVMI, FS, Rest EF & WMSI, peak EF & WMSI) parameters.	DSE is an effective means of risk assessment for insulin-dependent diabetics who are candidates for kidney and/or pancreas transplantation.
Poldermans, et al. (Am J Cardiol. 1996)	302 candidates for major vascular surgery.	Clinical and echocardiographic (Rest EF, peak EF, Peak WMA) parameters.	DSE is an effective means of risk assessment for perioperative complications of vascular surgery.
Cortigiani, et al. (J Am Soc Echocardiogr. 2017)	230 DM	Clinical and echocardiographic (rest EF, peak EF, CFR, LAD rest velocity, LAD peak velocity) parameters.	Ischemia was detected in 25% of patients included and was related to a poor outcome.
Chaowalit, et al. (J Am Coll Cardiol. 2006)	2349 DM	Clinical and echocardiographic (LVEF and RWMA) parameters.	Risk for MACE was stratified in three grades according to clinical features and the stress test performance.
D’Andrea, et al. (Eur J Echocardiogr. 2003)	325 DM	Clinical and echocardiographic (Rest EF & WMSI, peak EF & WMSI) parameters.	Detection of wall motion abnormalities and EF <40% during stress echocardiography determine poor prognosis in subjects with DM.
Oliveira, et al. (Cardiovascular Ultrasound 2009)	193 DM	Clinical and echocardiographic (Rest EF & WMSI, peak EF & WMSI) parameters.	Exercise echo is effective in determining prognosis of subjects with DM.
Cortigiani, et al. (Circ Cardiovasc Imaging 2015)	14,140 patients 2835 DM and 11,305 non-DM	Clinical and echocardiographic (Rest EF & WMSI, peak EF & WMSI, Contractile Reserve) parameters.	Detection of ischemia in all subjects referred for SE is a powerful prognostic factor of MACE. Antianginal treatment does not limit negative predictive value of SE in subjects with DM.
Bigi, et al. (Diabetes Care.2001).	259 DM	Clinical, echocardiographic (Rest EF & WMSI, peak EF & WMSI) and exercise parameters.	DSE is an efficient means of risk assessment in diabetics, superior to exercise ECG.
van der Sijde, et al. (J Am Soc Echocardiogr. 2017)	396 DM	Clinical and echocardiographic (Rest EF & WMSI, peak EF & WMSI, WMA) parameters.	DSE effectively predicted a favorable outcome up to seven years after the exam, only in patients who achieved the predicted for age maximum heart rate at peak stress.
Philouze, et al. (Diabetes Care. 2012)	44 patients with DM2 35 matched controls	Clinical and echocardiographic parameters (LVESV, LVEDV, LVEF, IVSEDD, LVPWEDD, LAVI, E/A ratio, DT, TDI parameters, DRWT, LS, CS, torsion and twist by STE).	While left ventricular mechanics at rest showed no significant difference between diabetics and controls, during stress test alterations occurred with regard to longitudinal strain, circumferential strain, apical, basal rotation and twist.

CAD: Coronary Artery Disease, CFR; Coronary Flow Reserve, DM: Diabetes Mellitus, DSE: Dobutamine Stress Echocardiography, EE: Exercise Echocardiography, EF: Ejection Fraction, LAVI: Left Atrial Volume Index, LV: Left Ventricular, LVIVSEDD: Interventricular Septal End-diastolic Dimension, LVEDD: Left Ventricular End-Diastolic Dimension, LVEDV: Left Ventricular End-Diastolic Volume, LVESD: Left Ventricular End-Systolic Dimension, LVESV: Left Ventricular End-Systolic Volume, LVMI: Left Ventricular Mass indexed to estimated body surface area, LVPW-EDD: Left Ventricular Posterior Wall End-diastolic Dimension, LVEF: Left Ventricular Ejection Fraction, NIDDM: Non-Insulin dependent Diabetes Mellitus, RWMA: Resting Wall Motion Abnormalities, SE: Stress Echocardiography, STE: Speckle Tracking Echocardiography, TTE: Transthoracic Echocardiography, WMA: Wall Motion Abnormalities, WMSI: Wall Motion Score Index.

**Table 4:** Studies referring to CFR in diabetics.

First Author/Study Name	Study Population	Features Studied	Endpoints
Kawata, et al. (Cardiovasc Diabetol. 2013)	135 DM2	Clinical, echocardiographic (LVEF, LVMI, CFR) and treadmill exercise parameters.	CFR has predictive role in DM type2 subjects and values < 2.5 are related with an adverse prognosis.
Nemes, et al. (Diabetes Res Clin Pract. 2007)	347 DM	Clinical, echocardiographic (LVEDV, LVEF, CFR, aortic atherosclerosis) and angiographic parameters.	Diabetics with defective microcirculation, as determined by declined CFR, are at high risk for cardiovascular events.
Atar, et al. (Echocardiography. 2012)	47 subjects (DM, preDM, controls)	Clinical and echocardiographic parameters (LVEDD, LVESD, LVEF, E/A, CFR, LA diameter).	CFR was diminished in diabetics. Prediabetics and normal controls had similar CFR, in normal range.
Ikonomidis, et al. (Atherosclerosis. 2015)	76 subjects 36 first degree relatives of DM, normal OGTT 20 normal OGTT, no family history of diabetes 20 abnormal OGTT.	Clinical, echocardiographic (TDI parameters, GLS, GL SRS & SRE, twisting, peak twisting and untwisting velocity by STE, CFR), Vascular (PWV, AI) and biochemical (OGTT-glucose and insulin levels) parameters.	First-degree relatives of DM with normal OGGT and subjects with abnormal OGTT had comparable resistance to insulin, defective arterial elastic properties and diminished CFR.
Yokoyama, et al. (Diabetes. 1998)	31 NIDDM & CAD 16 age-matched controls.	Clinical, echocardiographic, PET scan and biochemical parameters.	CFR was related to serum glucose regulation, as defined by HbA1c and fasting glucose.
Akasaka, et al. (J Am Coll Cardiol. 1997)	29 DM (18 diabetic retinopathy) 15 controls	Clinical, echocardiographic (LVEDV, LVESV, LVPW-EDD, IVS-EDD, LVEF, LVMI), angiographic (LVEDP, PCWP, Coronary diameter, CFR) and clinical parameters.	CFR is diminished in subjects with DM and the decrease is more remarkable in those with complications, such as diabetic retinopathy.

AI: Augmentation Index, CAD: Coronary Artery Disease, CFR; Coronary Flow Reserve, DM: Diabetes Mellitus, LVIVSEDD: Interventricular Septal End-diastolic Dimension, LVPWEDD: Left Ventricular Posterior Wall End-diastolic Dimension, LVEDD: Left Ventricular End-Diastolic Dimension, LVESD: Left Ventricular End-Systolic Dimension, LVEDV: Left Ventricular End-Diastolic Volume, LVESV: Left Ventricular End-Systolic Volume, LVEDP: Left Ventricular End-diastolic Pressure, LVEF: Left Ventricular Ejection Fraction, LVMI: Left Ventricular Mass indexed to estimated body surface area, NIDDM: Non-Insulin dependent Diabetes Mellitus, OGGT: oral glucose tolerance test, PCWP: Pulmonary Capillary Wedge Pressure, PWV: Pulse Wave Velocity, STE: Speckle Tracking Echocardiography TTE: Transthoracic Echocardiography.
